# Interactions between Egg Storage Duration and Breeder Age on Selected Egg Quality, Hatching Results, and Chicken Quality

**DOI:** 10.3390/ani10101719

**Published:** 2020-09-23

**Authors:** Hedia Nasri, Henry van den Brand, Taha Najar, Moncef Bouzouaia

**Affiliations:** 1 Laboratory LMMA and UCAR, Department of Animal Sciences, National Agronomic Institute of Tunisia, University of Carthage-Tunisia, 43 Avenue Charles Nicolle, Tunis 1082, Tunisia; najar.taha@inat.agrinet.tn; 2 Adaptation Physiology Group, Wageningen University, P.O. Box 338, 6700 AH Wageningen, The Netherlands; henry.vandenbrand@wur.nl; 3 National School of Veterinary Medicine, University of Manouba-Tunisia, Ariana, Sidi Thabet 2020, Tunisia; moncef.bouzouaia@ceva.com

**Keywords:** storage duration, breeder age, egg weight loss, hatchability, hatchling quality

## Abstract

**Simple Summary:**

Egg storage duration and breeder age are two important factors influencing productivity and profitability of hatcheries. These factors probably interact with each other to influence egg quality, apparent fertility, hatchability, and hatchling quality. The aim of this study was to investigate interactions between egg storage duration and broiler breeder age on these parameters. It was demonstrated that eggs from young breeders were the most resistant to storage duration increase in relationship to early and middle embryonic mortality than eggs from older breeders. However, the opposite was found for hatchling quality, where yolk free body mass, which increased from young to old breeders after five days of storage, increased only from middle to old breeders after prolonged storage (19 days). The intestine percentage decreased also after long storage in younger breeders, but in older breeders no significant effect of egg storage duration was found.

**Abstract:**

Egg storage duration and breeder age are probably interacting to influence egg quality, hatchability, and hatchling quality. To evaluate this interaction, the impact of breeder age (31, 42, 66 weeks) and storage duration (2, 5, 12, 19 days) was investigated on broiler breeder eggs (Arbor Acres). Thick albumen diameter and pH increased, and yolk dry matter decreased between 2 and 19 days of storage. With the increase of breeder age from 31 to 66 weeks, albumen height, percentage and dry matter and shell percentage decreased and the egg weight and yolk percentage, dry matter and diameter increased. Prolonged egg storage increased the yolk pH in all breeder ages, but earlier and steeper in the oldest breeders. Prolonged egg storage resulted in a lower hatchability of set and fertile eggs due to a higher percentage of embryonic mortality. Early mortality increased earlier and steeper with prolonged egg storage in the oldest compared to younger breeders. Between 5 and 19 days of storage, yolk free body mass, liver and proventriculus + gizzard percentages decreased, as well as hatchling length and yolk efficiency (yolk absorption per initial yolk weight). The latter effects were most pronounced in the younger than in the older breeders. Therefore, eggs are preferably stored shorter than 7 d, but if long storage (≥12 days) cannot be avoided, we recommend to store eggs of older breeders when egg quality and hatchability are most important. In case hatchling quality is most important, it would be better to store eggs of younger breeders (31 weeks) for a prolonged period.

## 1. Introduction

The quality of the day-old chickens and the subsequent broiler performance is affected by several factors, such as the breeder nutrition [1,2], strain, health status, age, environment (season, housing) [2,3,4], and preincubation factors (such as egg storage duration, storage temperature, turning, preincubation warming profile) [4,5,6,7] and incubation factors (such as temperature, CO_2_ concentration, oxygen availability, turning) [4,8,9].

In this study, two of these important factors (egg storage duration and breeder age) are investigated. Hatching eggs can be stored with little or no effects on hatchability until 4 to 7 days [10]. Prolonged egg storage (>7 days) has been shown to result in retarded egg quality parameters [11], higher embryonic abnormalities and mortality, lower hatchability [10,11,12] and poorer hatchling quality [13,14,15]. Additionally, to the effects of storage duration, also a higher breeder age negatively affects fertility [16], egg quality, early and late embryonic mortality [17,18,19] and hatchling weight [20,21].

Significant interactions between egg storage duration and breeder age have been observed as well, both on egg characteristics [18,22] and embryonic development [4,14,23]. The latter two studies reported that the decrease in embryonic growth rate, embryonic viability, and hatchability with prolonged storage duration was more pronounced with older breeders than in young breeders (59 vs. 32 weeks). Reis et al. [24] also reported a stronger negative effect of prolonged egg storage on embryonic viability and hatchability in broiler breeders of 48 to 50 weeks of age compared to broiler breeders of 32 to 34 weeks of age.

The differences in negative effects of prolonged egg storage with breeder age are probably related to changes in the embryo (e.g., number of viable cells) or egg characteristics (e.g., albumen pH and viscosity, shell quality), or in both of them [25,26]. As shown above, the interaction between storage duration and breeder age varies between studies; for review see Nasri et al. [27]. Additionally, in most studies only a limited number of breeder ages and storage durations were included. Consequently, additional studies to confirm or reject interactive effects of egg storage duration and breeder age on egg characteristics, embryonic development and hatchability are needed. The aim of this study was to investigate interactions between egg storage duration and broiler breeder age on egg quality, embryonic development and mortality, hatchability, and hatchling quality.

## 2. Materials and Methods

### 2.1. Experimental Design

**Figure 1:**
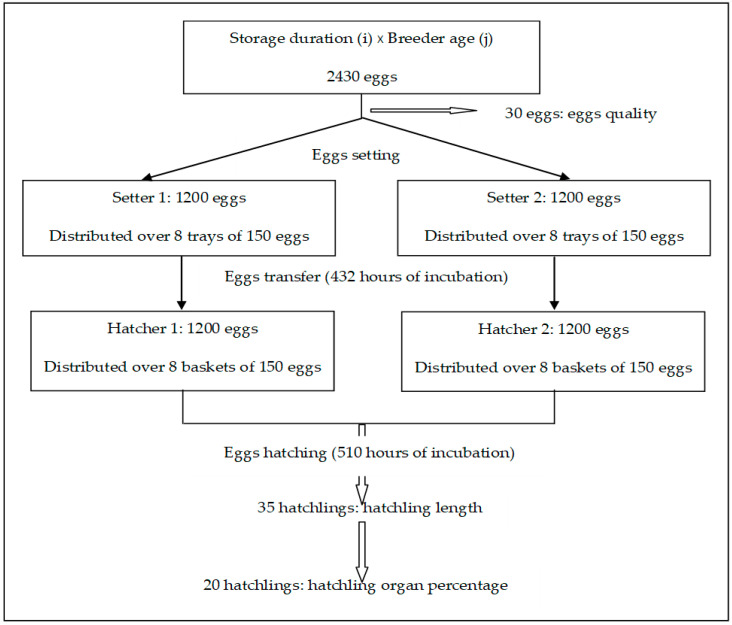
Random repetition scheme from one combination (treatment). Storage duration (i = 2, 5, 12 or 19 days), breeder age (j = 31, 42 or 66 weeks). Hatchling length and hatchling organ percentages were considered only with storage durations of 5 and 19 days.

The experiment was set up as a 4 × 3 factorial arrangement with four egg storage durations (2, 5, 12 and 19 days) and three broiler breeder ages (31, 42 and 66 weeks). Eggs of these storage durations and breeder ages were incubated together and egg characteristics and hatchability were determined. In part of the treatments (5 and 19 days of egg storage and 31, 42 and 66 weeks of breeder age (2 × 3 factorial arrangement)) additional measures were performed on hatchling quality. A random repetition scheme from one treatment was provided in Figure 1. The experimentation protocol was approved by the Animal Use and Care Committee of the National School of Veterinary medicine (E.N.M.V) of Tunisia (Number CEEA-EMNV 09/19).

### 2.2. Eggs and Egg Storage

In total 29,160 eggs were collected on the same day from three commercial breeder flocks (Arbor Acres; 9720 eggs per flock), which were reared at the same conditions as described by the management guide. Breeder ages were 31, 42 and 66 weeks. Collected eggs were stored in the hatchery in the same room and at the same conditions. The storage temperature was 16.5 °C and the relative humidity was 70%. Oxygen level and carbon dioxide level were not adjusted during storage. The experiment was performed at the Poulina hatchery, Saouef, Tunisia, between November and December 2017.

### 2.3. Egg Quality

After 2, 5, 12 and 19 days of storage, 30 eggs per breeder age and storage duration were sampled for egg characteristics (360 eggs in total). Egg characteristics of the 2 days stored eggs were used as the reference. Eggs were weighed and thereafter broken onto a flat surface, where thick albumen height, thick albumen diameter and yolk diameters (perpendicular to each other) were determined with an electronic digital caliper (Matrix, Guangdong, China). The height of the thick albumen was measured in the middle of the thick albumen equidistant from the outer edge of the albumen and the yolk [28]. The albumen and the yolk were then separated, and the fresh yolk weight was determined. Thereafter, the yolk and the albumen were homogenized separately, and their pH was measured immediately with an electronic pH meter (Ohaus, ST2100-F, Parsippany, NJ, USA). Shell weight was measured after washing and drying for 24 h at room temperature. The weight of the albumen was calculated as the difference between the weight of the egg and the weight of the yolk plus the shell. Albumen and yolk were then dried at 105 °C to constant weight for dry matter (DM) determination [29].

### 2.4. Incubation

The rest of the eggs (28,800 eggs) were divided into 192 incubation trays of 150 eggs each. Each tray contained eggs of one breeder age and one storage duration, corresponding to a total egg of 2400 eggs per storage duration and breeder age (16 trays per treatment). Eggs were stored in the same room on incubation trolleys. After 2, 5, 12 and 19 days of storage, 16 trays per breeder age were incubated. Trays were divided over four incubation trolleys with four trays per breeder age per trolley. Trays of the different breeder ages were arranged alternatively on trolleys. The two trays at the bottom and the two trays at the top of each trolley were not part of the experimentation. The four trolleys per storage duration were divided over two identical Petersime incubators (BioStreamer™, Zulte, Belgium, capacity of 57,600 eggs) with 2 trolleys per incubator. Experimental trolleys were placed as the first trolleys near the fan. Each incubator was further filled with other eggs (10 trolleys), which were not part of the experimentation. All incubators were set at an incubation temperature of 37.8 °C (at the start of incubation), which temperature declined to 36.1 °C at the end of day 17 of incubation. Relative humidity was maintained between 74% and 94%. Carbon dioxide level was maintained below 0.85%. Eggs were turned hourly at an angle of 90°.

At 18 days of incubation, all eggs were transferred into hatching crates and moved to hatchers. The same order of trays applied in setters was applied for hatching crates in the hatchers. For each egg storage duration two Petersime hatchers (BioStreamer™, Zulte, Belgium, capacity of 19,200 eggs) were used. In each one, experimental trolleys were placed as the first trolleys near the fan. Each hatcher was further filled with other eggs (2 trolleys), which were not part of the experimentation. All hatchers were set at an incubation temperature 36.7 °C (at the start of the hatcher phase), which temperature declined to 35.6 °C at the end of incubation. Relative humidity was maintained between 82% and 89%. Carbon dioxide level was maintained below 1.00%.

At 510 h after the start of incubation, hatchlings were collected. The total number of hatchlings as well as the number of saleable hatchlings (clean, dry, and free from deformities: no skin lesions, well-formed beak, normal conformation of legs, completely sealed navel, and no yolk sac or residual membrane protruding from the navel area) according to Tona et al. [15], was counted per hatching basket. The non-saleable chickens were considered as second grade chickens.

Unhatched eggs were opened and macroscopically examined to determine apparent fertility or the stage of embryonic mortality. Embryonic mortality was classified into three groups: early mortality (from 1 to 7 days), middle mortality (from 8 to 17 days) and late mortality (from 18 to 21 days). Embryonic mortality is expressed as percentage of fertile eggs. Fertility was calculated as: (number of fertile eggs/number of incubated eggs) × 100%. Hatchability of set eggs was calculated as: (number of hatched chickens/number of incubated eggs) × 100%. Hatch of fertile was calculated as: (number of hatched chickens/number of fertile eggs) × 100%. Fertility and hatchability were calculated per tray/hatching basket, which was used as the experimental unit.

### 2.5. Hatchling Quality

Hatchling length and Pasgar score [13] of 35 hatchlings were determined for 5 and 19 days of storage and for all 3 breeder ages. Hatchlings were chosen randomly from each hatching basket, with 2 to 3 hatchlings per basket. Thereafter, 20 hatchlings per breeder age and for storage duration 5 and 19 days were killed by cervical dislocation and body weight (BW), residual yolk weight and organ (heart, liver, intestines, and stomach (gizzard plus proventriculus)) weight were determined. Yolk free body mass (YFBM) was calculated as BW minus residual yolk weight. The yolk efficiency was calculated as: (yolk efficiency = [(yolk weight at set − residual yolk weight at hatch)/yolk weight at set] × 100. To calculate the yolk efficiency, we used the average of fresh yolk weight measured per treatment (storage duration × breeder age). Organ percentages were expressed as weights relative to YFBM.

### 2.6. Statistical Analysis

Data were processed using the statistical software SAS version 9.1 (2004). For each parameter, distribution of means and residuals were examined to verify model assumptions. In case data were not normally distributed, a log transformation was performed. For egg quality parameters, hatchling weight, hatchling length and organ weights a generalized linear model (GLM) was performed. The experimental unit for egg parameters was the egg. The experimental unit for hatchling weight, hatchling length and organs weight was the hatchling. The model used for these variables was:

(1) Y= μ+Storage duration+Breeder age+Interaction+e, 

where *Y* = dependent variable, *µ* = overall mean, *Storage duration* = *storage duration* (2, 5, 12, or 19 days), *Breeder age* = Breeder flock age (31, 42 or 66 weeks of age), *Interaction* = interaction between storage duration and breeder flock age, *e* = residual error.

For fertility and hatchability data, the experimental unit was the tray/hatching basket. The data were analyzed with a mixed model. The model used for these variables was:

(2) Y= μ+Storage duration+Breeder age+Interaction+Incubator+e, 

where *Y* = dependent variable, *µ* = overall mean, *Storage duration* = *storage duration* (2, 5, 12, or 19 days), *Breeder age* = Breeder flock age (31, 42 or 66 weeks of age), *Interaction* = interaction between storage duration and breeder flock age, *Incubator* = incubator (1, 2), e = residual error. Trolley was considered as a random factor.

For the Pasgar score, to assess the multinomial results a logistic procedure was used. The experimental unit was the hatchling. Data were expressed as LSmeans ± SEM. LSmeans were compared using Bonferroni adjustments for multiple comparisons. Significance was based on *p* ≤ 0.05.

## 3. Results

### 3.1. Egg Quality

An interaction between breeder age and storage duration was found for albumen (*p* = 0.006) and yolk (*p* = 0.003) pH (Table 1). For all storage durations, except for 19-day storage duration, the highest albumen pH was found for the 42-week breeders, followed by 66-week and finally 31-week breeder flock. Yolk pH did not differ between breeder ages for storage durations 2, 12 and 19 days, whereas for 5-day stored eggs, yolk pH was higher in 66-week breeders than in 31-week breeders, with 42-week breeders in between and not different from both other ages.

Thick albumen diameter (*p* < 0.001) and yolk diameter (*p* < 0.001) increased with storage duration (Table 1). The increase of the albumen and the yolk diameters was faster for the first days of storage than thereafter, resulting in a lack of difference between 12 and 19 days of storage. Shell percentage was higher after 5 days of storage than after 19 days of storage (*p* < 0.05), with both other days in between and not different from the other storage durations.

With the increase of the breeder age from 31 to 66 weeks, egg weight increased (*p* < 0.001; Table 1). The yolk percentage increased as well with breeder age (*p* < 0.001), but albumen percentage decreased (*p* < 0.001). Shell percentage decreased (*p* < 0.05) from 31 to 66 weeks. Yolk and thick albumen diameter increased as well with breeder age (*p* < 0.001), whereas the albumen height decreased (*p* < 0.001).

Yolk DM percentage showed an interaction between breeder age and storage duration (Table 2). After 2 and 5 days of storage, yolk DM increased (*p* < 0.05) from 31 to 66 weeks breeder flocks, but this did not happen after 12 and 19 days of storage. Alternatively explained, the yolk DM percentage decreased (*p* < 0.05) with storage duration for 42- and 66-week breeder age (Table 2). The decrease started earlier (from 5 days) with the 42-week compared to the 66-week breeder age (12 days). However, with the 31-week breeders, no influence of storage duration on the yolk DM percentage was found.

The albumen DM percentage decreased (*p* < 0.001) with breeder age.

### 3.2. Hatching Results

An interaction between breeder age and storage duration was found for early (*p* < 0.001) and middle (*p* = 0.05) mortality (Table 3). For early mortality, in 12- and 19-day stored eggs, it was higher in 66-week old breeders than in 31- and 42-week old breeders (*p* < 0.05), whereas in 2- and 5-day stored eggs no effect of breeder age was found. For middle mortality, no effect of breeder age was found in 2-, 5- or 12- day stored eggs, whereas in 19-day stored eggs, middle mortality was higher in the 66-week breeders than in the 31- and 42-week breeders (*p* < 0.05).

Apparent fertility was lower in the 19-day stored eggs than in the 5- and 12-day stored eggs (*p* < 0.05), with 2-day stored eggs in between. Hatchability of set eggs was lowest in the 19-day stored eggs, followed by the 12- and 2-day stored eggs (*p* < 0.05). Five-day stored eggs had higher hatchability of set eggs (*p* < 0.05) than 12-day stored eggs with the 2-day stored eggs in between. Hatchability of fertile eggs was lower (*p* < 0.05) in the 19-day stored eggs than in the other three storage durations.

Apparent fertility (*p* < 0.05), hatchability of set eggs (*p* < 0.05) and hatchability of fertile eggs (*p* < 0.05) were lower in 66-week breeders than in 31- and 42-week breeders. Late mortality was higher in the 66-week broilers than in the 31- and 42-week broilers (*p* < 0.5).

### 3.3. Hatchling Quality

An interaction between egg storage duration and breeders age was found for hatchling weight (*p* = 0.005), YFBM (*p* < 0.001) and intestine percentage (*p* = 0.01) (Table 4). Hatchling weight (*p* < 0.05) and YFBM (*p* < 0.05) increased with breeder age from 31 to 66 weeks in 5-day stored eggs, whereas in 19-day stored eggs, this only occurred from 42 to 66 weeks of age. For intestine percentage, in 31-week breeder age, intestine percentage was lower in 19-day stored eggs than in 5-day stored eggs, whereas in 42- and 66-week breeder age, no effect of storage duration was found (*p* < 0.05).

Eggs stored for 19 days resulted in hatchlings with higher residual yolk weight (*p* < 0.001), lower liver percentage (*p* < 0.001), shorter hatchlings (*p* < 0.001) and lower yolk efficiency (*p* < 0.001) compared to hatchlings originating from eggs stored for 5 days.

Breeders of 66 weeks of age resulted in hatchlings with more residual yolk (*p* < 0.05), lower heart percentage (*p* < 0.05) and lower Pasgar score (*p* < 0.05) than breeders of 31 and 42 weeks of age. Additionally, hatchling length increased (*p* < 0.001) with breeder age.

## 4. Discussion

### 4.1. Egg Characteristics

Egg albumen pH and yolk pH showed an interaction between storage duration and breeder age. With the increase of storage duration, albumen pH increased for all breeder ages, but for the youngest breeder age this increase was the smallest. For yolk pH, effects of storage duration were small, but earlier in the oldest breeders and largest in the young breeders. Consequently, the difference between albumen and yolk pH was smallest in the youngest breeders. This is in accordance with earlier findings [18,22] and can be explained by the lower albumen quality already at oviposition in older breeders [30] and the lower albumen buffering capacity with an increase in breeder age [31]. This lower buffering capacity is related to the easier CO_2_ loss from the albumen [32], which is related to a more rapid release of CO_2_ through the eggshell. This phenomenon is the consequence of a higher eggshell conductance (due to its lower eggshell quality) in eggs from older hens [33,34] that results from the decrease of the eggshell thickness with the increase of the breeder age [26]. The lower buffering capacity will finally result in faster increase of albumen pH [35].

Besides interaction effects, the main effects of storage duration and breeder age were also found. The deterioration of the egg quality by the increase of the storage duration was reported by many authors. This deterioration was revealed by an increase of the albumen pH and yolk pH with the increase of storage duration. The present results agree with Yuceer et al., [36] who noted an increase of the fresh albumen pH of broiler eggs from 8.64 to 9.03 after 21 d of storage, with Şekeroğlu et al. [37], who noted an increase of the fresh albumen pH of layer eggs from 8.62 to 9.28 after 21 days of storage and with Kouame et al. [38] who noted an increase of the albumen pH of Guinea fowl eggs from 9.64 after 3 days of storage to 10.17 after 11 days of storage. Sheng et al., [39] also reported an increase in yolk pH from 6.10 in fresh eggs to 6.32 and 6.43 in stored eggs (50 days) at 4 and 22 °C, respectively. This pH increase was attributed to the carbon dioxide loss from the breakdown of carbonic acid in egg yolks [40]. The egg quality deterioration was also revealed by the increase of thick albumen and yolk diameter [41,42]. For example, Khatun et al. [40] reported an increase (+2.42 mm) of the thick albumen diameter with 7 days of storage. In fact, when eggs are stored for a prolonged period, the ovomucin layer, which is responsible for the firmness of thick albumen, becomes weaker. The albumen, therefore, spreads over a wider area, which causes an increase of the albumen length and width and consequently the decrease of the albumen height [41].

Changes in yolk diameter are probably the result of the weakening of chalazae and vitelline membrane with prolonged storage. These chalazae hold the yolk in position and absorb shocks and jerks to eggs [43]. With long storage duration of eggs, the yolk vitelline membrane gets ruptured more easily, causing the yolk to lose its round shape. Finally, the yolk becomes fragile, flattened and eventually gets mixed up with albumen [41]. The weakening of the vitelline membrane, which is probably related to the increase of the albumen pH, facilitates the migration of water from the albumen to the yolk [44]. Consequently, yolk DM will decrease [45], which has been shown in the current study as well, particularly in older breeders. Due to the dilution of the yolk, its consistency decreases, resulting in a more flattened yolk with a larger diameter.

The increase of the breeder age had a large spectrum of influences on the egg quality. It resulted in an increase of all egg components weights, but at different degrees. The increase of the yolk weight, which was accompanied with the increase of the yolk DM, was more pronounced compared to that of the albumen. Relatively, only yolk percentage increased, whereas albumen and shell percentage decreased. This is in agreement with Nangsuay et al. [3] who reported that albumen and yolk percentage change happened independently of the egg weight and Yadgary et al., [46] who noted an increase of the yolk weight by more than 40% compared to an increase by 13.3% for albumen weight, in eggs from 50 weeks compared to 30 weeks of breeder age. This apparent discrepancy between increase in yolk weight and albumen weight with increasing breeder age is probably related to the increase of ovulation intervals with the increase in breeder age [47]. With longer ovulation intervals, the same amount of yolk from hepatic synthesis is deposited in a lower number of follicles [48], resulting in larger yolk weights with higher DM percentage at ovulation. The current study joins findings of Ahn et al. [49], Suarez et al. [50] and Ulmer-Franco et al. [51] who noted an increase of yolk DM and a decrease of albumen DM with the increase of the breeder age. For example, Ahn et al. [49] noted an albumen and yolk DM rate of 12.7% and 50.7% at 28 weeks vs. 11.8% and 51.6% at 97 weeks of breeder age, respectively. Nangsuay et al. [3] also noted with a Ross-308 strain, a higher DM rate in the albumen of eggs from 29 weeks compared to 53 weeks breeder age (0.132 vs. 0.120 g dry basis/g wet weight).

### 4.2. Hatching Results

Early and middle mortality increased with storage duration as well as breeder age. An interaction between storage duration and breeder age was also found for these two parameters. In fact, prolonged storage resulted in a higher early and middle mortality than shorter storage, but this effect was more pronounced in older breeders. Okur et al. [52] and Özlü et al. [53] also noted a higher embryonic mortality rate with stored eggs from older breeders (55 vs. 32 weeks and 27 vs. 50 weeks, respectively). Tona et al. [18] attributed the higher reduction of hatchability of prolonged stored eggs of older breeders to the lower quality of the albumen at oviposition compared to younger breeders. The blastoderm is located adjacent to the albumen and consequently, changes in the viscosity or pH of the albumen may play an integral role in determining the viability of the embryo during the very early stages of development [28]. Alternatively, the higher embryonic mortality can also be attributed to differences in the embryonic developmental stage at oviposition. A more advanced developmental stage with storage duration in both young (32 weeks) and old breeders (63 weeks) resulted in a similar developmental stage after 28 days of storage [4]. These results might partly explain the difference between young and old breeders on the impact of short storage and the similarity between results of different breeder ages with longer egg storage duration. Therefore, it can be suggested that it is important to investigate the very early embryonic stage (cellular scale) of stored eggs from old and young breeders to understand how each flock is reacting to storage.

Short egg storage (5 days) resulted in higher hatchability than egg storage of 19 days. Results are in line with those of Pokhrel et al. [4] who noted in young breeders that hatchability was higher after 7 days of storage than after 0 days of storage and decreased with longer storage duration (21 to 28 days) in both young and old breeders. A higher hatchability noted with short storage in young breeders can be explained by the rise of albumen pH with storage duration associated with a decrease of albumen height and viscosity. Albumen liquefaction probably facilitates the movement of nutrients from the albumen to the blastoderm [54] and can reduce resistance to gaseous diffusion through the albumen [55]. When incubation already starts before liquefaction, thus without egg storage, oxygen or metabolite acquisition may be insufficient to meet the metabolic demands of the early chick embryo, which may result in higher mortality [28]. This assumption is only valid for young breeders, characterized by a high albumen viscosity, whereas in older breeders with already a deteriorated albumen quality at oviposition, CO_2_ is already lost quite easily. Other authors also suggested that some dormancy (4 to 7 days) is beneficial for embryonic viability during the first stages of incubation [54,56,57].

The negative impact of prolonged storage duration on hatchability was reported by several authors [4,58,59,60]. It can be explained in part by morphological changes in the blastoderm [60,61,62] with increased cell apoptosis and necrosis [63] during storage. In fact, a decline of the total blastodermal cell counts with the increase of storage duration was noted by Uyanga et al. [60] with Marshall^®^ broiler breeders and by Cai et al. [64] on Blue-breasted quail breeders. Bakst and Akuffo [65] reported that storage for 5 days of turkey eggs already altered the macroscopic and microscopic appearances of blastoderms. Visually, blastoderms were both slightly asymmetric and enlarged. Histologically, blastoderms from stored eggs had lower or no subgerminal cavities and cells comprising the epiblast appeared to be compacted with some cell nuclei exhibiting pyknotic or apoptotic characteristics. Due to these cellular and morphological changes, it can be speculated that during early incubation the embryo is not able to start cell division properly or will start cell division with some delay after the start of incubation, resulting in more embryonic mortality or malformation of embryos.

Obtained results revealed that storage duration affects mostly the early embryonic development. Once the embryo succeeds to pass the critical developmental stage, it will pursue its development normally. This suggests that early embryogenesis, taking place within the first 3 days of incubation, is the critical stage for embryonic survival after prolonged storage. This can reflect the significant changes occurring during storage to the embryo itself and to its surrounding environment (the yolk and the albumen) [4]. Schmidt et al. [10] noted also that the highest impact of increasing storage was particularly due to the early mortality (0 to 6 days) with 40.32%, followed by the late mortality (18 to 21 days) with 37.61% recorded after 14 days of storage.

Hatchability results were comparable between the 32- and 42-week breeder ages. In the old breeders (66 weeks), monitored parameters were deteriorated. These results are consistent with other studies [8,66,67]. The fertility decrease can be explained by the decline of the ability of older hens to retain spermatozoa in the utero-vaginal sperm host gland (UV-SHG) as observed in broiler breeders [68] and turkeys [69,70] and by the lower mating activity of the roosters [71]. It might also be explained by a lower quality of follicles, which cannot be fertilized [68]. First sequence eggs in a clutch also showed lower hatchability than later sequence eggs and these eggs are more frequent in aged breeders. Fasenko et al. [68] suggested that with the extended period of time of the follicle of a first sequence egg spent on the ovary, follicle-aging takes place, which might result in yolk compositional differences. All these factors might have played a role in the lower hatchability in older breeders.

The rate of late dead embryos also increased with 66-week breeder age. This fact can probably be explained by difficulties in losing embryonic metabolic heat during the latter stages of incubation and consequently a higher embryo temperature [72,73] for embryos from older breeders. In fact, these embryos have higher heat production, because older breeders produce larger eggs with more nutrients, resulting in a higher metabolic rate [3] than younger breeders.

### 4.3. Hatchling Quality

Egg storage duration interacted with breeder age on hatchling weight, YFBM and intestine percentage. Hatchlings from the middle age breeders (42 weeks) were the most impacted by egg storage duration and those from the oldest breeders (66 weeks) were the most resistant.

Prolonged storage declined hatchling quality by decreasing its length and decreasing the percentage of the liver and intestines. Hatchling length has been suggested to be an indicator of later life broiler performances [74], although others did not show a strong relationship between hatchling length and later life performances [75]. Prolonged storage also resulted in higher residual yolk sac weight and lower YFBM and yolk efficiency, meaning that prolonged storage reduced the ability of embryos to absorb yolk during incubation. These results agree with those of Reijrink et al. [5], who noted that hatchlings after 4 days of storage were 0.1 cm longer and had 0.5 more grams of YFBM than hatchlings after 14 days of storage. Yalcin et al. [58] also reported a decrease by −1.6 g of the hatchling weight when the storage duration was extended from 3 d to 14 d. Goliomytis et al. [76] noted also, that 1-day-old chick BW and length were linearly negatively correlated with egg storage length. It can be suggested that due to the lower yolk sac utilization (also reflected in a lower yolk efficiency), less energy is available for the developing embryo and less energy can be deposited in organs, resulting in lower organ percentages. It can be speculated that due to prolonged egg storage, the vitelline membrane is retarded (see above) and consequently the development of the yolk sac membrane is retarded as well. This might have consequences for absorption capacity of the yolk sac membrane. The higher residual yolk weight, noted with chickens from long stored eggs, can also be, in part, a result of the hatch delay noted with storage [15]. In fact, hatchlings were all pulled at the same moment (510 h after start incubation) and consequently, hatchlings from short stored eggs, which hatched earlier, were exposed to a longer period between hatching and pulling than hatchlings from prolonged stored eggs. This difference in hatching time probably contributes to the lower residual yolk sac weight with hatched chickens from short stored eggs (5 days) compared to long stored eggs. Concerning the YFBM decrease with prolonged egg storage, Hamidu et al. [61] suggested that this is due to a reduction in blastodermal cell numbers even before the eggs were incubated. This phenomenon is the result of the increase of necrotic and late apoptotic-necrotic cells with the increase of the egg storage duration (14 d vs. 4 d).

The increase of the breeder age resulted in higher hatchling weight, YFBM and residual yolk sac weight, lower heart percentage, longer hatchlings, lower Pasgar score and higher yolk efficiency. These differences were mostly due to the breeders of 66 weeks, whereas breeders of 31 and 42 weeks hardly differed from each other. The increase of the residual yolk weight with breeder age can probably partly be explained by the higher yolk weight at set. These results agree those of Latour et al. [77] who studied hatchlings from Arbor Acres breeder aged of 36 and 51 weeks. Damaziak et al. [78] noted also, a decrease of the chick quality (−5 points based on Tona score) as well as an increase of the chick weight (+9.2 g) with breeder age (49 to 52 weeks vs. 70 to 73 weeks). In the same line, Rifkhan et al. [79] reported the increase of the hatchling weight by +12.72 g and the hatchling length by +1.1 cm when breeder age increased from 26–35 weeks to 46–55 weeks, which is probably related to egg weight at set. Koppenol et al. [80] also noted an increase of the liver weight (+0.23 g) and percentage (+0.08%) at hatch with the increase of the breeder age (from 28 to 48 weeks). In the same line, Sinclair et al. [81] reported that hatchlings originating from older breeders were usually larger and of higher quality compared to smaller hatchlings from young breeders, because they are naturally more resistant to dehydration upon hatching. It can be suggested that the higher YFBM with older breeders is related to the higher ability of the embryo to absorb yolk during incubation, due to the larger yolk sac membrane and vascular system of the larger yolk of older breeder compared to the smaller yolk of younger breeder [82]. Another explanation might be the higher energy content of eggs from older breeder hens, especially through the increase of yolk size, than eggs from younger breeder. Because egg energy used during incubation was positively related to yolk size, embryos of old breeder eggs transferred more energy into chicken YFBM than those of the young breeder eggs [3].

## 5. Conclusions

It can be concluded that both storage duration and broiler breeder age affect egg composition, hatching characteristics, and hatchling quality, but for a number of these characteristics an interaction between storage duration and breeder age was found. Prolonged egg storage (≥12 days) has a larger negative impact on egg quality and hatchability of eggs from old breeders (66 weeks) than on eggs of young breeders (31 and 42 weeks). However, the opposite was found for hatchling quality, where effects of prolonged egg storage (≥19 days) appear to be larger in younger breeders (31 and 42 weeks) than in older breeders (66 weeks).

## Figures and Tables

**Table 1:** Effects of egg storage duration and broiler breeder age and their interaction on egg characteristics at the start of incubation (LSMeans ± SEM).

	Egg Weight, g	Albumen Height, mm	Yolk Diameter, mm	Thick Albumen Diameter, mm	Yolk, % ^1^	Albumen, % ^1^	Shell, % ^1^	Albumen pH	Yolk pH
Storage (day)									
2	63.9	7.87	42.9 ^c^	81.2 ^c^	31.0	60.3	8.91 ^ab^	9.14	6.14
5	64.0	7.69	43.7 ^b^	88.5 ^b^	31.2	59.7	9.11 ^a^	9.22	6.16
12	63.8	7.59	44.1 ^ab^	91.7 ^ab^	31.7	59.5	8.86 ^ab^	9.32	6.20
19	64.7	7.45	44.3 ^a^	95.4 ^a^	31.3	59.8	8.83 ^b^	9.42	6.22
SEM	0.4	0.1	0.2	1.1	0.2	0.2	0.07	0.01	0.01
Age (week)									
31	58.8 ^c^	8.34 ^a^	41.4 ^c^	82.6 ^c^	29.5 ^c^	61.3 ^a^	9.13 ^a^	9.21	6.18
42	62.8 ^b^	7.69 ^b^	43.6 ^b^	89.6 ^b^	30.9 ^b^	60.1 ^b^	9.14 ^a^	9.33	6.17
66	70.7 ^a^	6.91 ^c^	46.2 ^a^	95.4 ^a^	33.5 ^a^	58.0 ^c^	8.52 ^b^	9.29	6.19
SEM	0.4	0.1	0.1	1.0	0.2	0.2	0.06	0.01	0.01
Storage × age									
2 × 31	58.3	8.33	40.9	76.3	29.3	61.6	9.05	9.08 ^g^	6.13 ^d^
2 × 42	63.2	8.34	42.9	80.4	31.2	59.9	9.26	9.20 ^e^	6.15 ^bd^
2 × 66	70.2	6.95	44.8	86.9	32.4	59.2	8.42	9.14 ^f^	6.14 ^cd^
5 × 31	59.1	8.47	41.2	80.7	29.1	61.5	9.35	9.15 ^f^	6.14 ^cd^
5 × 42	62.7	7.70	43.3	87.1	30.7	60.0	9.25	9.28 ^d^	6.15 ^bcd^
5 × 66	70.1	6.91	46.5	97.7	33.7	57.6	8.72	9.24 ^e^	6.20 ^ab^
12 × 31	58.4	8.40	41.6	85.6	30.0	60.9	9.12	9.23 ^e^	6.21 ^a^
12 × 42	62.5	7.35	44.3	94.9	30.9	60.0	9.04	9.39 ^b^	6.18 ^ad^
12 × 66	70.6	7.01	46.5	94.5	34.1	57.5	8.43	9.33 ^c^	6.19 ^ac^
19 × 31	59.5	8.18	42.0	87.6	29.7	61.3	9.00	9.37 ^bc^	6.24 ^a^
19 × 42	62.6	7.38	44.0	96.1	30.6	60.4	9.00	9.45 ^a^	6.20 ^ab^
19 × 66	72.0	6.78	47.0	102.7	33.7	57.8	8.50	9.45 ^a^	6.21 ^a^
SEM	0.7	0.2	0.3	1.9	0.4	0.4	0.12	0.01	0.01
*p*-values									
Storage	0.46	0.23	<0.001	<0.001	0.16	0.11	0.03	<0.001	<0.001
Age	<0.001	<0.001	<0.001	<0.001	<0.001	<0.001	<0.0001	<0.001	0.08
Storage × age	0.70	0.43	0.11	0.10	0.09	0.17	0.76	0.005	0.003

a–g: LSmeans within a column and factor lacking a common superscript differ (*p* ≤ 0.05). ^1^ As percentage of egg weight.

**Table 2:** Effects of egg storage duration and broiler breeder age and their interaction on albumen and yolk dry matter (DM) percentage at the start of incubation (LSmeans ± SEM).

	Albumen DM, % ^1^	Yolk DM, % ^1^
Storage (day)		
2	12.78	50.30
5	12.79	49.78
12	12.80	49.28
19	12.80	48.78
SEM	0.06	0.12
Age (week)		
31	13.25 ^a^	48.48
42	12.86 ^b^	49.74
66	12.26 ^c^	50.37
SEM	0.05	0.10
Storage × age		
2 × 31	13.32	48.52 ^dg^
2 × 42	12.84	50.93 ^a^
2 × 66	12.19	51.44 ^a^
5 × 31	13.26	48.05 ^fg^
5× 42	12.73	49.96 ^b^
5 × 66	12.38	51.32 ^a^
12 × 31	13.20	48.84 ^cdf^
12 × 42	12.99	49.46 ^bdg^
12 × 66	12.21	49.54 ^bc^
19 × 31	13.22	48.53 ^dg^
19 × 42	12.90	48.62 ^ceg^
19 × 66	12.28	49.19 ^bde^
SEM	0.10	0.20
*p*-values		
Storage	1.00	<0.001
Age	<0.001	<0.001
Storage × age	0.39	<0.001

a–g: LSmeans within a column and factor lacking a common superscript differ (*p* ≤ 0.05). ^1^ DM = dry matter; calculated as (albumen or yolk DM weight/fresh albumen or yolk weight) × 100.

**Table 3:** Effects of egg storage duration, broiler breeder age and their interaction on apparent fertility, hatchability and embryonic mortality (means ± SE).

	Apparent Fertility, % ^1^	Hatchability of Set, % ^2^	Hatchability of Fertile, % ^3^	Early Mortality (Day 1–7), %	Middle Mortality (Day 8–17), %	Late Mortality (Day 18–21), %
Storage (day)						
2	89.0 ^ab^	84.7 ^ab^	90.91 ^a^	2.39	0.73	0.85
5	92.4 ^a^	85.7 ^a^	92.97 ^a^	3.16	0.46	2.67
12	90.9 ^a^	83.5 ^b^	92.37 ^a^	4.12	1.35	1.60
19	87.8 ^b^	74.1 ^c^	85.71 ^b^	8.94	1.89	2.94
SEM	1.1	0.5	1.26	0.72	0.21	0.36
Age (week)						
31	97.1 ^a^	91.0 ^a^	94.10 ^a^	3.31	0.72	1.24 ^b^
42	96.6 ^a^	89.9 ^a^	93.73 ^a^	3.04	0.85	1.40 ^b^
66	76.3 ^b^	65.0 ^b^	83.64 ^b^	7.90	1.82	3.68 ^a^
SEM	0.7	0.5	1.1	0.65	0.19	0.30
Storage × age						
2 × 31	96.0	93.4	95.58	2.32 ^a^	0.77 ^b^	0.52
2 × 42	95.8	93.2	95.09	2.89 ^a^	0.17 ^b^	0.52
2 × 66	75.2	67.5	82.06	3.51 ^ab^	0.69 ^b^	2.18
5 × 31	99.5	94.0	95.11	2.19 ^bdj^	0.77 ^b^	1.69
5 × 42	98.4	93.2	95.29	2.31 ^bdj^	0.60 ^b^	1.72
5 × 66	79.5	70.0	88.52	4.97 ^cdk^	0.89 ^b^	4.59
12 × 31	97.6	93.0	95.69	2.24 ^bj^	0.17 ^b^	1.21
12 × 42	97.2	91.2	94.98	2.68 ^bj^	0.34 ^b^	0.69
12 × 66	77.9	66.2	86.42	7.44 ^c^	0.87 ^b^	2.91
19 × 31	95.4	83.8	90.02	6.96 ^c^	0.67 ^b^	1.61
19 × 42	95.2	82.3	89.56	5.59 ^cj^	1.31 ^b^	2.85
19 × 66	72.8	56.3	77.54	14.28 ^k^	2.05 ^a^	4.36
SEM	1.3	0.9	2.2	0.86	0.27	0.52
*p*-values						
Storage	<0.001	<0.001	<0.001	<0.001	<0.001	0.33
Age	<0.001	<0.001	<0.001	<0.001	<0.001	<0.001
Storage × day	0.57	0.50	0.39	<0.001	0.05	0.21

a–k: LSmeans within a column and factor lacking a common superscript differ (*p* ≤ 0.05); ^1^ Apparent fertility = (number of fertile eggs/number of incubated eggs) × 100%; ^2^ Hatchability of set eggs = (number of hatched chickens/number of incubated eggs) × 100%; ^3^ Hatchability of fertile = (number of hatched chickens/number of fertile eggs) × 100%.

**Table 4:** Effects of egg storage duration, broiler breeder age and their interaction on chicken quality characteristics and organs weight at hatching (LSmeans ± SEM).

Effect	Hatchling Weight, g	Residual Yolk Weight, g	YFBM, g ^1^	Heart, % of YFBM	Liver, % of YFBM	Intestine, % of YFBM	Stomach, % of YFBM	Hatchling Length, cm	Pasgar Score	Yolk Efficiency, % ^2^
Storage (day)										
5	44.82	5.14 ^b^	39.69	0.88	4.00 ^a^	4.96	6.83 ^a^	20.29 ^a^	9.8	74.3 ^a^
19	44.99	7.18 ^a^	37.79	0.87	3.55 ^b^	4.44	6.50 ^b^	19.87 ^b^	9.7	64.9 ^b^
SEM	0.41	0.20	0.30	0.01	0.05	0.07	0.08	0.04	0.1	0.93
Age (week)										
31	40.87	5.83 ^b^	35.04	0.90 ^a^	3.84	4.71	6.48	19.60 ^c^	9.9 ^a^	66.6 ^a^
42	44.00	5.60 ^b^	38.40	0.90 ^a^	3.79	4.79	6.79	20.17 ^b^	9.9 ^a^	70.8 ^b^
66	49.83	7.04 ^a^	42.79	0.84 ^b^	3.69	4.6	6.71	20.47 ^a^	9.5 ^b^	71.5 ^b^
SEM	0.50	0.24	0.37	0.01	0.06	0.09	0.09	0.05	0.1	1.15
Storage × Age										
5 × 31	40.32 ^a^	4.63	35.70 ^a^	0.91	4.02	5.18 ^a^	6.63	19.83	9.9	73.1
5 × 42	45.28 ^b^	4.75	40.53 ^b^	0.90	4.00	5.01 ^ab^	7.07	20.37	10.0	75.3
5 × 66	48.86 ^cd^	6.03	42.83 ^c^	0.83	3.98	4.69 ^ac^	6.78	20.68	9.7	74.4
19 × 31	41.41 ^a^	7.03	34.38 ^a^	0.88	3.66	4.24 ^c^	6.34	19.37	9.9	60.1
19 × 42	42.71 ^ac^	6.45	36.26 ^a^	0.90	3.58	4.57 ^bc^	6.51	19.98	9.8	66.2
19 × 66	50.79 ^d^	8.05	42.74 ^c^	0.84	3.40	4.51 ^bc^	6.64	20.27	9.4	68.5
SEM	0.72	0.34	0.53	0.02	0.09	0.12	0.13	0.08	0.6	1.62
*p*-values										
Storage	0.81	<0.001	<0.001	0.61	<0.001	<0.001	<0.001	<0.001	0.42	<0.001
Age	<0.001	<0.001	<0.001	<0.001	0.24	0.31	0.07	<0.001	<0.001	0.007
Storage × Age	0.005	0.59	<0.001	0.51	0.47	0.01	0.28	0.89	0.11	0.11

a–d: LSmeans within a column and factor lacking a common superscript differ (*p* ≤ 0.05). ^1^ YFBM weight = BW - residual yolk weight. ^2^ Yolk efficiency = ((yolk weight at set − residual yolk weight at hatch)/yolk weight at set) × 100.
